# Supplementary material for: Prevalence of Venous Thromboembolism in Intensive Care Units: A Meta-Analysis
Source: J Clin Med. 2022 Nov 11;11(22):6691. doi: 10.3390/jcm11226691 (PMC9698016; doi:10.3390/jcm11226691)
Supplement: Supplementary file 1 [file jcm-11-06691-s001.zip › jcm-1992480-supplementary.pdf]

Journal of
Clinical Medicine

MDPI

Supplementary

Study name	Point	Lower limit	Upper limit	Z-Value	p-Value
	Statistics with study removed				
Akaraborworn (2016)	0.11	0.08	0.15	-10.71	0.00
Dibiasi (2022)	0.11	0.08	0.15	-10.57	0.00
Wilsey (2019)	0.11	0.07	0.15	-10.57	0.00
Salim (2009)	0.11	0.07	0.15	-10.75	0.00
Okumus (2015)	0.11	0.07	0.15	-10.58	0.00
Tachino (2019)	0.11	0.07	0.15	-10.56	0.00
Muci?o-Bermejo (2015)	0.10	0.07	0.15	-10.59	0.00
Khouli (2006)	0.11	0.07	0.15	-10.55	0.00
Ow (2021)	0.11	0.07	0.15	-10.55	0.00
Tucker (1973)	0.10	0.07	0.15	-10.57	0.00
Park (2009)	0.10	0.07	0.15	-10.63	0.00
Kollef (1998)	0.10	0.07	0.15	-10.57	0.00
Karcutskie (2017)	0.10	0.07	0.15	-10.61	0.00
Malinoski (2013)	0.10	0.07	0.15	-10.56	0.00
Adimi Naghan (2013)	0.10	0.07	0.15	-10.60	0.00
Obi (2015)	0.10	0.07	0.15	-9.90	0.00
Chu (2021)	0.10	0.07	0.15	-10.46	0.00
Muscedere (2007)	0.10	0.07	0.15	-10.51	0.00
Harahsheh (2019)	0.10	0.07	0.15	-10.61	0.00
Karcutskies (2017)	0.10	0.07	0.15	-10.36	0.00
Nickele (2013)	0.10	0.07	0.15	-10.65	0.00
Patel (2013)	0.10	0.07	0.15	-10.62	0.00
Barton (2022)	0.10	0.07	0.15	-10.66	0.00
Brendan Cloney (2019)	0.10	0.07	0.15	-10.30	0.00
Praeger (2012)	0.10	0.07	0.15	-10.70	0.00
Allen (2016)	0.10	0.07	0.15	-10.56	0.00
Brandi (2020)	0.10	0.07	0.14	-10.65	0.00
Phan (2022)	0.10	0.07	0.14	-10.61	0.00
Abdel (2022)	0.10	0.07	0.14	-10.59	0.00
Camous (2014)	0.10	0.07	0.14	-10.72	0.00
Dhillon (2021)	0.10	0.07	0.14	-10.71	0.00
Gibson (2020)	0.10	0.07	0.14	-10.66	0.00
Ravenel (2009)	0.10	0.07	0.14	-10.72	0.00
Valle (2014)	0.10	0.07	0.14	-10.77	0.00
Thorson (2012)	0.10	0.07	0.14	-10.79	0.00
Beitland (2019)	0.10	0.07	0.14	-10.81	0.00
Hamada (2017)	0.10	0.07	0.14	-10.83	0.00
Fu (2018)	0.10	0.07	0.14	-10.83	0.00
Yumoto (2017)	0.10	0.07	0.14	-10.84	0.00
Minet (2012)	0.10	0.07	0.14	-10.85	0.00
Mei (2022)	0.10	0.07	0.13	-12.41	0.00
Patel (2005)	0.09	0.07	0.13	-12.38	0.00
	0.10	0.07	0.14	-10.82	0.00

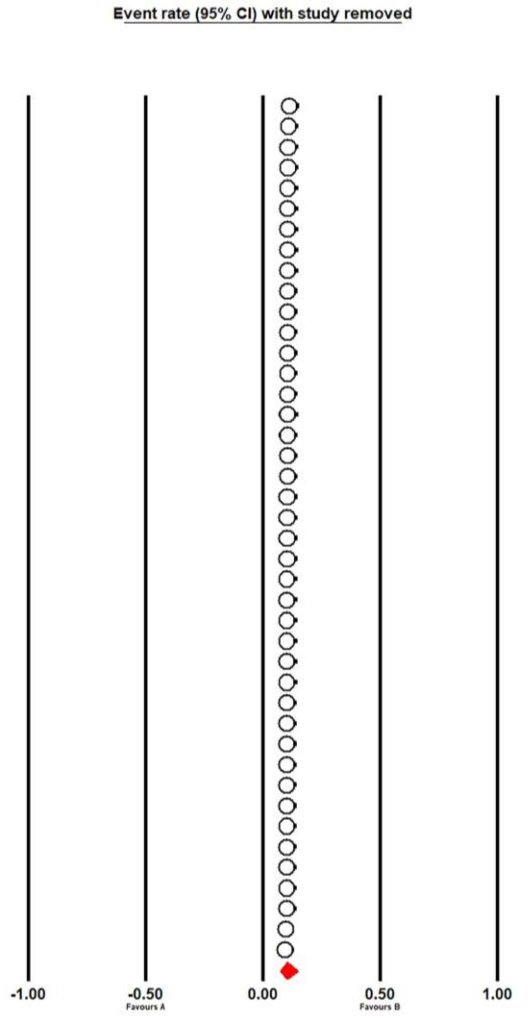

Figure S1. Sensitive analysis on prevalence of VTE in intensive care patients [5,6,25–64]. The red symbol indicated the prevalence of VTE in ICU patients.

Table S1. Metaregression analyses of prevalence of VTE in intensive care patients.

Continuous variable	B Value (Point Estimate)	Z Value	p Value
Age	0.04455	11.99	$p < 0.001$
Study quality	−0.09251	−3.36	$p < 0.001$
Sample size	−0.00030	−22.41	$p < 0.001$
Malignancy	−0.01705	−8.23	$p < 0.001$
Sex	−0.00984	−4.08	$p < 0.001$
Spinal cord injury	0.07058	7.45	$p < 0.001$
ISS	0.16487	12.99	$p < 0.001$
